# Safety and efficacy of tacrolimus-coated silicone plates as an alternative to mitomycin C in a rabbit model of conjunctival fibrosis

**DOI:** 10.1371/journal.pone.0219194

**Published:** 2019-07-05

**Authors:** Sam Young Yoon, Eun-Soon Kim, Gill Sang Han, Leejee H. Suh, Hyun Suk Jung, Hungwon Tchah, Jae Yong Kim

**Affiliations:** 1 Department of Ophthalmology, Kangdong Sacred Heart Hospital, Hallym University College of Medicine, Seoul, Republic of Korea; 2 Edward S. Harkness Eye Institute, Columbia University Medical Center, New York, New York, United States of America; 3 Department of Ophthalmology, University of Ulsan College of Medicine, Asan Medical Center, Seoul, Republic of Korea; 4 School of Advanced Materials Science & Engineering, Sungkyunkwan University, Suwon, Republic of Korea; University Hospitals Cleveland, UNITED STATES

## Abstract

**Purpose:**

To find safer and more effective drugs than mitomycin C to prevent conjunctival fibrosis in a rabbit model.

**Methods:**

Twenty-four rabbits were involved and randomly divided into four groups. Limbus-based peritomy was performed at the superior cornea, and normal saline (NS group), mitomycin C (MMC group), SR (SR group), or TC (TC group)-coated silicone plate was inserted at the sub-Tenon’s space in each group. Conjunctival congestion was evaluated at 1 and 4 weeks postoperatively. At 4 weeks, the numbers of inflammatory cells, fibroblasts, myofibroblasts, blood vessels, and goblet cells were counted in the conjunctiva and Tenon’s capsule around the silicone plate.

**Results:**

At 4 weeks, conjunctival congestion was significantly less than that observed at 1 week in the SR and TC groups (*p* < 0.05), whereas the number of myofibroblasts was significantly lower in the MMC and TC groups (*p* < 0.05). The conjunctiva was significantly less congested in the TC group versus the other groups at 1 week and 4 weeks (*p* < 0.05). The TC group had the lowest number of inflammatory cells and MMC group had the lowest number of goblet cells among all groups (*p* < 0.05).

**Conclusions:**

The TC-coated silicone plate was more effective in inhibiting inflammation and fibrosis versus the MMC-coated silicone plate and was associated with fewer adverse effects in the rabbit model.

## Introduction

Conjunctival fibroblast may excessively transdifferentiate into myofibroblasts during wound healing after surgery for the treatment of glaucoma and recurrent pterygium. Severe conjunctival fibrosis is the most common cause of failure of trabeculectomy [1, 2]. Mitomycin C (MMC) is commonly used to effectively suppress the fibrotic reaction surrounding the wound and prevent excessive fibrosis. However, due to a potent antimetabolic reaction, use of MMC is related to many adverse effects including scleritis, necrotic keratitis, corneal edema, endothelial cell loss, and fibrotic encapsulated bleb [3–8]. Therefore, the discovery of effective alternative therapeutic options to control the wound healing response with fewer adverse effects compared with MMC is warranted. Thus far, efforts were focused on the identification of an alternative drug to MMC. In 2009, postoperative subconjunctival injection of the anti-vascular endothelial growth factor bevacizumab improved success and limited the formation of scar tissue after trabeculectomy in rabbits [9]. In 2010, the antifibrotic activity of bevacizumab in human Tenon’s fibroblasts was reported *in vitro* [10]. Tenon’s fibroblasts are key cells in the initiation and mediation of subconjunctival wound healing and formation of fibrotic scars [11]. Following the release of platelet-derived growth factor and transforming growth factor beta in large amounts at the wound site, the former activates fibroblast proliferation, whereas the latter stimulates fibroblast proliferation and myofibroblast differentiation [12–14].

In this study, we used a drug-coated silicon plate to investigate the effectiveness of sirolimus (SR) and tacrolimus (TC) in inhibiting fibrosis around the keratoconjunctival wound. Moreover, the safety profile of these agents versus MMC was assessed.

## Materials and methods

### Drug-coated silicon plates

**Fig 1:**
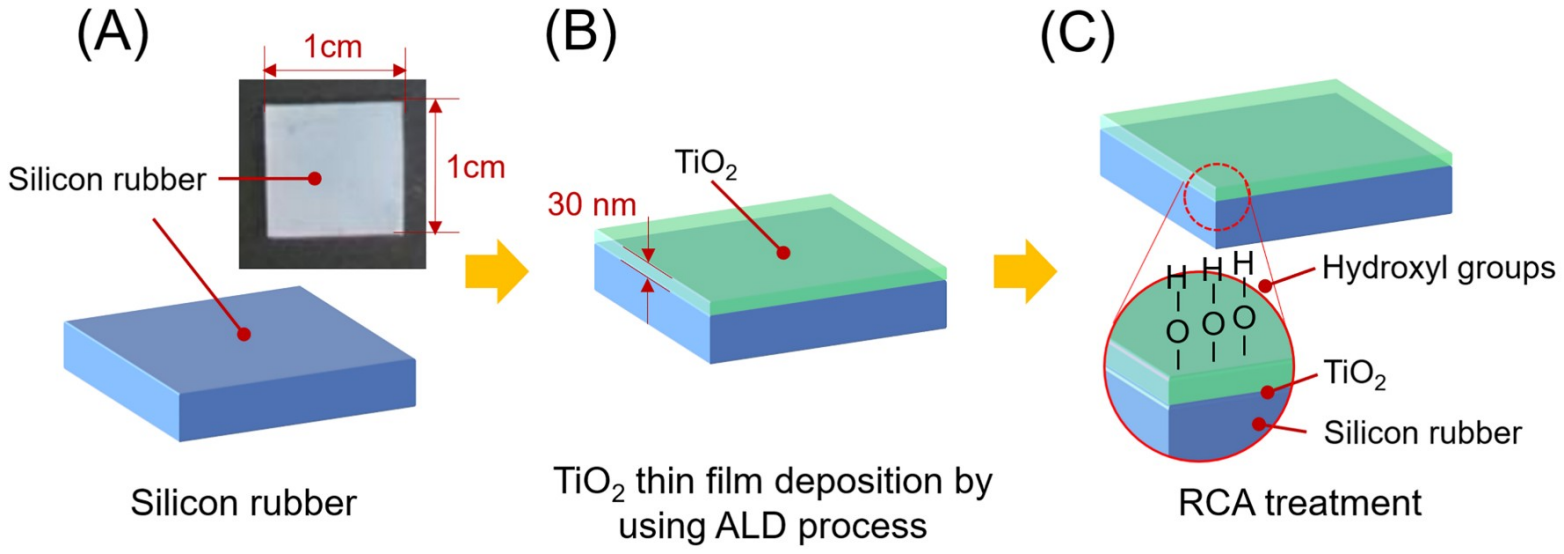
Creation of drug-coated implants. (A) An adequately sized silicone plate was prepared. (B) After titanium dioxide was hydrophilic coated up to a 30-nm thickness on the surface of the silicone plate using atomic layer deposition, the silicone plate could be coated with the candidate drugs using the nanometer scale. (C) RCA treatment, i.e., washing with hydrogen peroxide and ammonia aqueous solution, was performed before target drug treatment to eliminate cations present in the air and to retain the OH radical. The silicone plate was effectively coated with the target drugs. ALD = atomic layer deposition, RCA = Radio Corporation of America.

The drug-coated silicon plate was prepared by a co-researcher team in the School of Advanced Materials Science & Engineering and Department of Energy Science led by Professor Chung HS. In this study, silicone rubber—a polymer composed of silicon, carbon, oxygen, and hydrogen—exhibited very poor coating ability of the drug dispersed in a hydrophilic solvent. To overcome this problem, titanium dioxide (TiO_2_) was deposited as a thin film (thickness: 30 nm) on the surface of the silicone rubber. This TiO_2_ deposition allowed the hydrophilic surface coating (semi-conducting) to improve the coating ability of the drug using the atomic layer deposition (ALD) method. The coating mechanism of ALD involved chemical bonding and oxidation of the metal precursor at the substrate followed by the deposition of the drugs at a low temperature, unlike the conventional vapor deposition method. Although the substrate had a varying three-dimensional shape (e.g., silicone plates), the coating drug could be homogeneously deposited on the substrate. The OH radical formed at the deposited thin TiO_2_ film through the Radio Corporation of America (RCA) cleaning method improved the hydrophilic property and enhanced the drug-coating capability (Fig 1). The RCA clean, a standard set of wafer cleaning steps, has to be performed before high-temperature processing of silicon wafers in semiconductor manufacturing [15].

**Fig 2:**
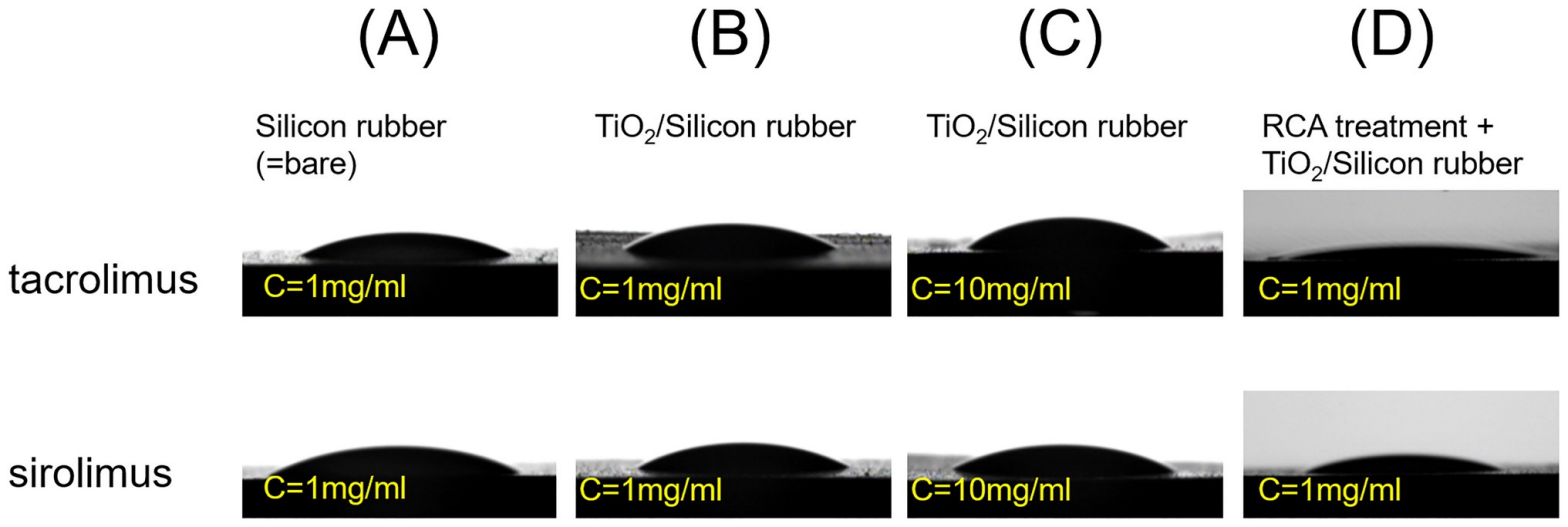
Contact angles of dissolved drugs on the silicon plate with different drug concentrations and coating methods. Larger contact angles of dissolved drugs indicate greater thickness of the drugs and vice versa. (B and C) When the concentration of the drug increased, the contact angle increased. (D) The contact angle on the silicon rubber with TiO_2_ thin film after RCA treatment was the smallest among all types of silicon rubber. The thin coating was caused by its hydrophilic property. C = concentration.

Following the addition of TC (FK-506, Enzo Life Sciences, Inc., Farmingdale, NY, USA) or SR (rapamycin, LC Laboratories, Inc., Woburn, MA, USA) on the silicon rubber with the TiO_2_ thin film after RCA treatment, the contact angle between the silicone rubber and a drug dissolved in ethanol was measured to evaluate wettability. The contact angle was reduced by >50% versus that observed on bare silicone rubber (Fig 2A and 2D, Table 1). These results suggest that the TiO_2_ thin film after RCA treatment rendered the surface of the silicone rubber hydrophilic. The contact angle increased in parallel with the concentration of the drug (Fig 2B and 2C, Table 1). Therefore, it was important to determine the appropriate drug concentration.

**Table 1:** Contact angles of dissolved drugs on the silicon with different drug concentrations and coating methods.

	Silicon rubber (C = 1 mg/ml)	TiO_2_/Silicon rubber (C = 1 mg/ml)	TiO_2_/Silicon rubber (C = 10 mg/ml)	RCA treatment + TiO_2_/Silicon rubber (C = 1 mg/ml)
Tacrolimus	30.1°	28.5°	38.6°	14.9°
Sirolimus	41.5°	30.5°	34.5°	17.8°

**Fig 3:**
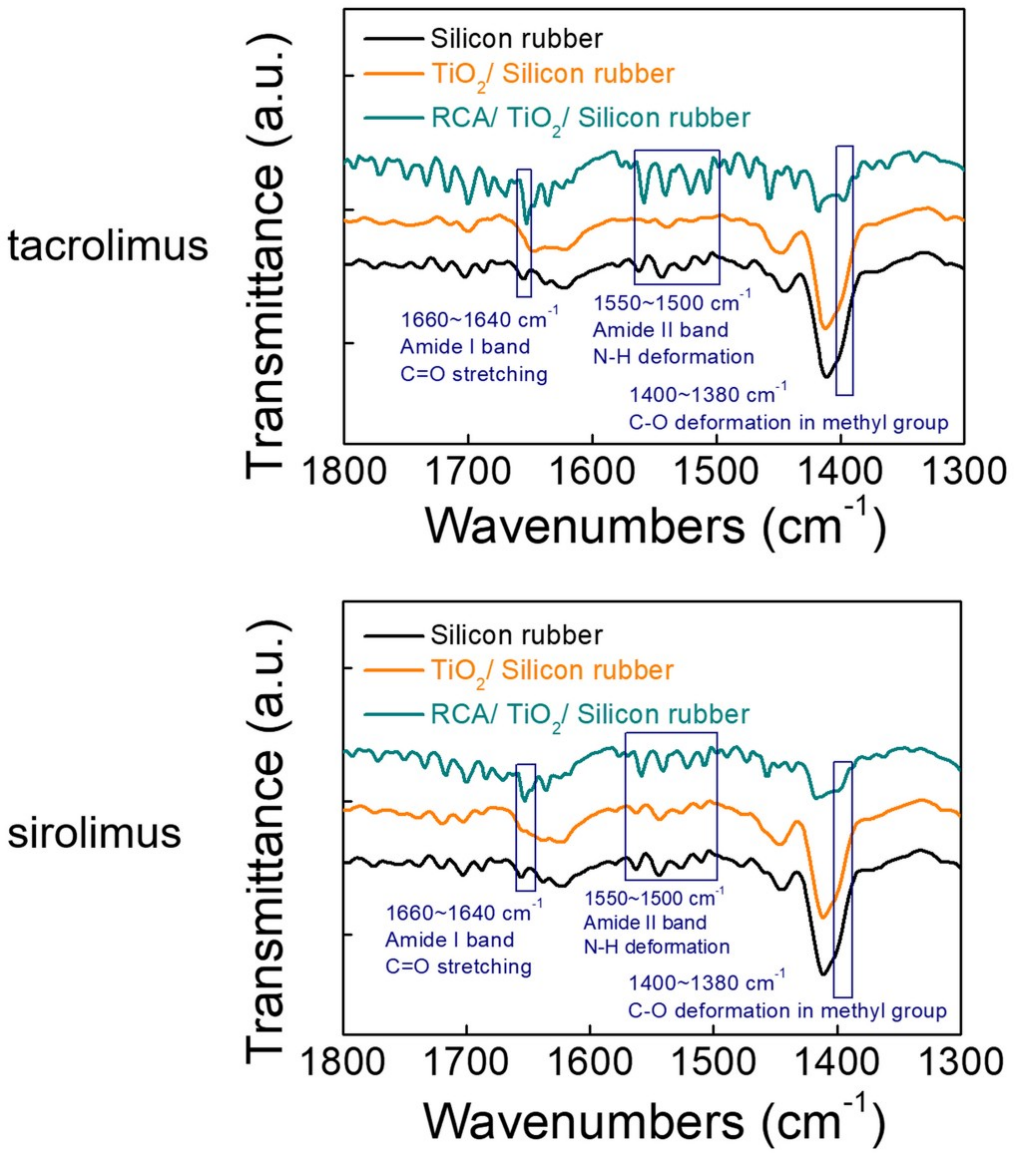
Transmittance of the drug-coated silicone plate.

The molecular structures of both SR and TC are mainly composed of methyl groups, C = O, C-O, and C-N bonding. Use of Fourier-transform infrared spectroscopy permitted to observe the coating properties. In addition, Fourier-transform infrared spectroscopy was used to measure transmittance and observe the effects of wettability on the coating ability of the drug after spin-coating of either 1 mg/mL TC or SR on the silicon plate. C-O deformation in the methyl group, N-H deformation, and C = O stretching appeared at 1380 cm^−1^, 1500–1550 cm^−1^, and 1640 cm^−1^, respectively (Fig 3). The intensity of the transmittance peak was found to gradually increase as follows: silicon rubber, silicone rubber with ALD coating, and RCA cleaning (Fig 3). Consequently, strengthening of the wetting properties improved the coating properties. After ALD coating and RCA cleaning, the silicon plates were effectively coated with the two drugs.

**Fig 4:**
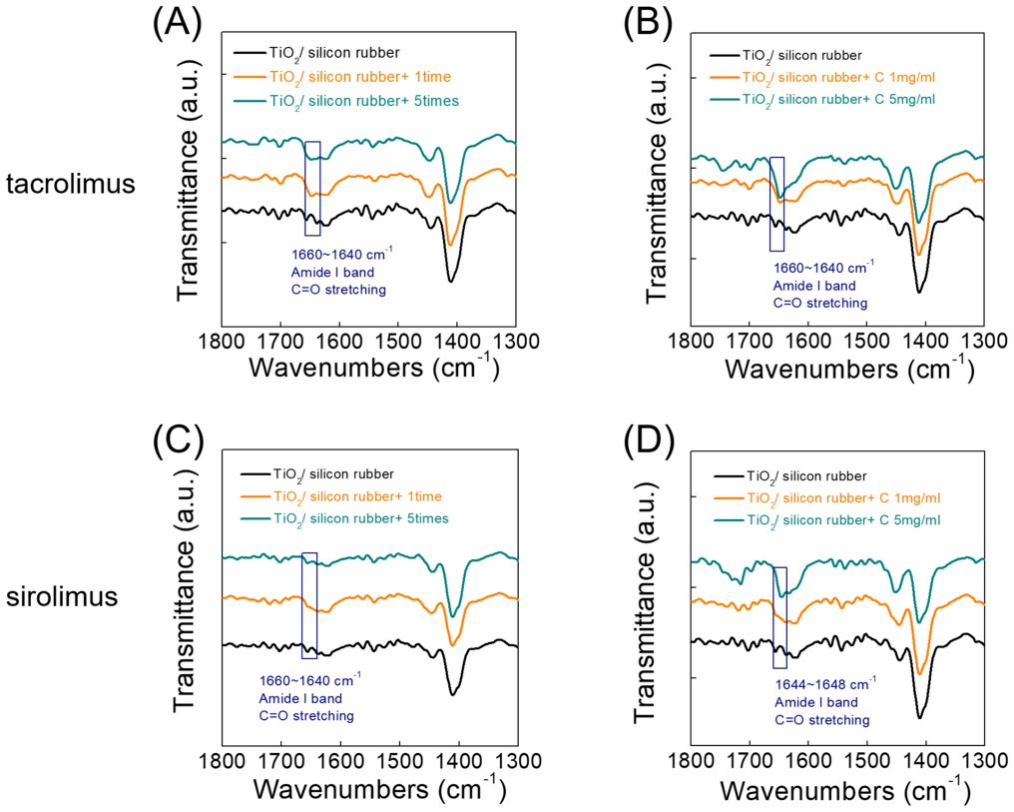
Change in transmittance of the drug-coated silicone plate according to the different coating conditions. (A) When tacrolimus (1 mg/ml) was repeatedly coated five times on the silicone plate, it had higher transmittance than single coated tacrolimus. (B) Tacrolimus coated at a higher concentration had higher transmittance. (C and D) The sirolimus-coated silicon plate showed the same properties.

Comparison of the TC 1 mg/mL spin-coated five times versus the TC 5 mg/mL spin-coated once on the silicone plate showed that the latter was more effective in increasing the intensity of the transmittance peak (Fig 4). This tendency in intensity was observed regardless of the type of drug.

Each drug-coated silicon plate was used in the animal experiments; 0.5% MMC (Kyowa Hakko Kirin Co. Ltd., Tokyo, Japan), 0.5% TC, 0.5% SR, and 0.9% saline solution as control.

### Animal Ethics

All procedures involving animals in this study conformed to the guidelines of the Association for Research in Vision and Ophthalmology concerning the use of animals in research. All rabbits were raised in individual cages and maintained under standard conditions. The experimental protocol was approved by the Institutional Animal Care and Use Committee of the Asan Medical Center, Seoul, Korea. (Permit Number: 201212168). Only one eye of each rabbit included in the study groups was subjected to the standard preparation for surgery, anesthesia, and surgical technique. All surgeries and interventions were performed under anesthesia, and all efforts were made to minimize suffering. After the rabbits were sacrificed by CO_2_ inhalation, death was confirmed.

### Study groups and surgical technique

Twenty-four healthy eyes of 24 New Zealand white male rabbits (aged 4–5 months and weighing 2.0–2.6 kg) were randomly divided into four groups (6 rabbits per group). All procedures were performed under general anesthesia induced through intramuscular administration of a mixed solution of Zoletil 50 (Tiletamine 125 mg+Zolazepam 125 mg/5cc, Virbac, Inc., Seoul, Korea, 10–20 mg/kg) and Rompun (Xylazine HCl 23.3 mg/mL, Bayer, Inc., Seoul, Korea, 2.2 mg/kg). The animal model of conjunctival fibrosis used in the study had been previously established by inserting a silicone plate at the sub-Tenon’s space of rabbits [16]. All procedures were performed by a single researcher (Yoon SY) to ensure consistency. After the application of 0.5% (wt/vol) proparacaine hydrochloride (Alcaine; Alcon-Couvreur, Puurs, Belgium) to achieve topical anesthesia, a 3 mm peritomy was made parallel to the limbus at the superior fornix 8 mm away from the superior limbus. An 8 × 8 mm sub-Tenon’s space was dissected between the Tenon’s capsule and sclera using Westcott scissors. The circular drug-coated silicone plate (5 mm diameter and 0.5 mm thickness) was inserted at the sub-Tenon’s space in each group as follows: 0.9% normal saline (NS group), 0.5% MMC (MMC group), SR (SR group), and TC (TC group). The silicon plate was fixed 2 mm away from the superior limbus using two 10–0 Nylon (Ethicon Inc., Somerville, NJ, USA) sutures. The peritomy incision was closed using an 8–0 coated Vicryl (Ethicon Inc.) running suture. Topical antibiotic ointment (0.3% Ofloxacin; Tarivid, Santen Pharmaceutical Co., Osaka, Japan) was instilled in the operated eye immediately after surgery. Topical antibiotic ointments were applied in the operated eyes thrice daily for 1 week after surgery.

### Histological preparation and assessment

All the animals were anesthetized as described above, and the anterior segment was evaluated using a light microscope (Leica Stereozoom S6 D, Leica, Wetzlar, Germany) and a portable slit-lamp biomicroscope (SL-15, Kowa, Nagoya, Japan) at 1 and 4 weeks after surgery. The degree of conjunctival congestion, status of conjunctival fibrosis, and the occurrence of complications (i.e., local infection, endophthalmitis, and implant exposure) were recorded. In addition, digital photographs of the conjunctiva around the inserted silicone plate were obtained at 32× magnification using a digital camera (Coolpix 4500, 4 megapixels; Nikon Imaging Japan, Tokyo, Japan) attached to a microscope to evaluate conjunctival congestion objectively at 1 and 4 weeks. The degree of conjunctival congestion was scored in the digital photographs obtained at 1 and 4 weeks using the Modified MacDonald–Shadduck Scoring System [17].

At 4 weeks, the silicone plates were carefully removed under anesthesia to minimize tissue damage. After euthanasia using CO_2_, all examined eyes were enucleated. The entire procedure after enucleation was conducted by the comparative pathology core lab at the Asan Medical Center, Seoul, Korea. The tissues were fixed for 48 hours with Davidson’s fixative (BBC Biochemical, Seattle, WA, USA). The tissues containing conjunctiva, Tenon’s capsule, and sclera around the inserted silicone plate were obtained, and paraffin blocks were subsequently produced using these tissues. After hematoxylin & eosin staining, inflammatory cells, fibroblasts, and goblet cells in the tissues were counted using light microscopy under a high-power field (400× magnification). Subsequently, staining with α-smooth muscle actin was performed to assess the density and distribution of myofibroblasts. The average values were repeatedly recorded in the three different fields of view by a skilled analyst. A double-blinded, randomized analysis was performed to reduce selection bias.

### Statistical analysis

Data were analyzed using the SPSS software (version 19.0; SPSS, Chicago, IL, USA). Statistical analyses between groups were performed using the Kruskal–Wallis test and the Games–Howell adjustment. The paired Wilcoxon signed-rank test was used to compare the values between weeks 1 and 4 after surgery. A *p* < 0.05 denoted statistical significance.

## Results

In all groups, conjunctival hyperemia tended to decrease at 4 weeks versus 1 week. The most severe conjunctival congestion was observed in the NS group, whereas the TC group exhibited the lowest degree of congestion (S1 Fig).

**Fig 5:**
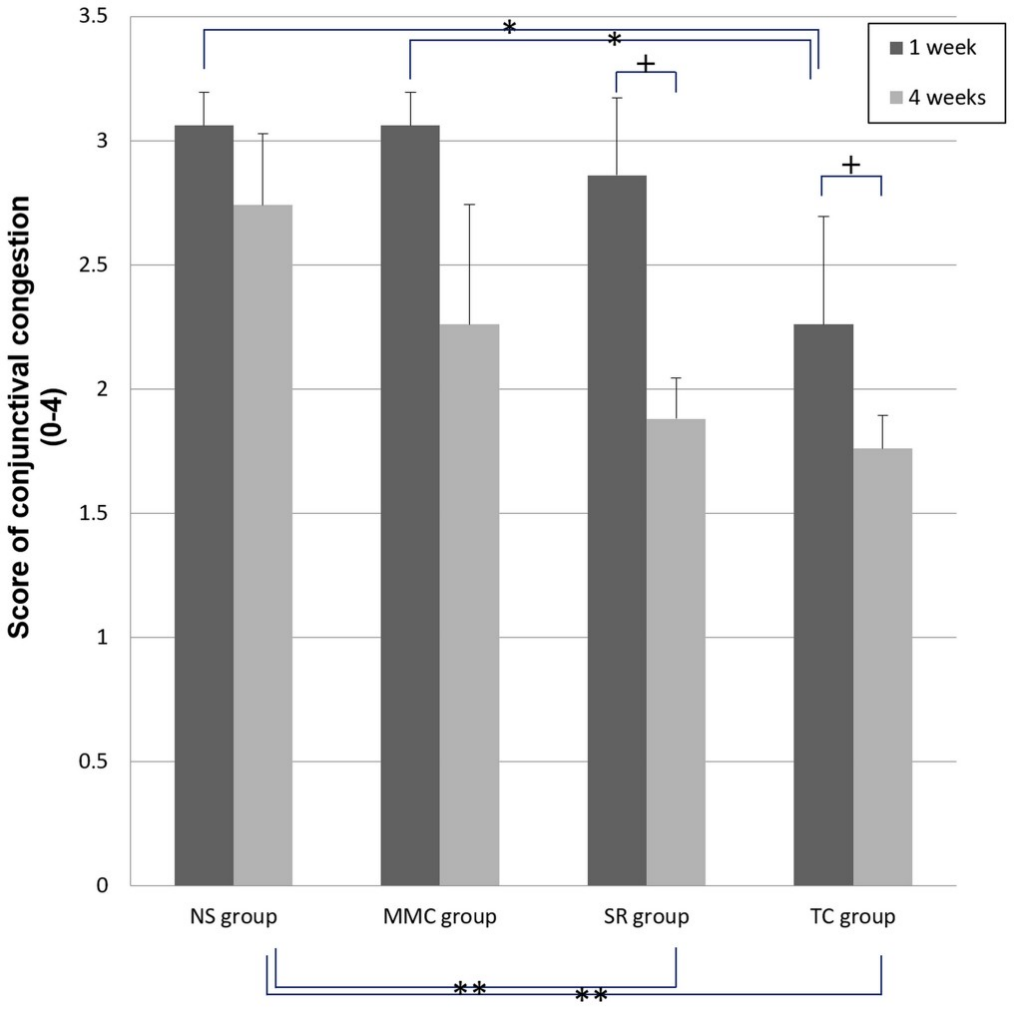
Conjunctival congestion in the rabbit at 1 and 4 weeks postoperatively. * = significant difference in conjunctival congestion at 1 week among all groups (*p* < 0.05, Kruskal–Wallis test and Games–Howell adjustment), ** = significant difference in conjunctival congestion at 4 weeks among all groups (*p* < 0.05, Kruskal–Wallis test and Games–Howell adjustment), † = significant difference between 1 and 4 weeks postoperatively (*p* < 0.05, Wilcoxon signed-rank test). NS = 0.9% normal saline, MMC = 0.5% mitomycin C, SR = sirolimus, TC = tacrolimus.

At 1 week after surgery, the TC group presented less conjunctival congestion than the NS and MMC groups (all, *p* < 0.05; Fig 5). At 4 weeks, there was less congestion observed in the SR and TC groups than in the NS group (*p* < 0.05; Fig 5). At 4 weeks, the degree of conjunctival congestion was significantly decreased in the SR and TC groups compared with that observed at 1 week (*p* < 0.05, Fig 5). Notably, conjunctival congestion did not differ between the TC and SR groups at 1 and 4 weeks (*p* > 0.05).

**Fig 6:**
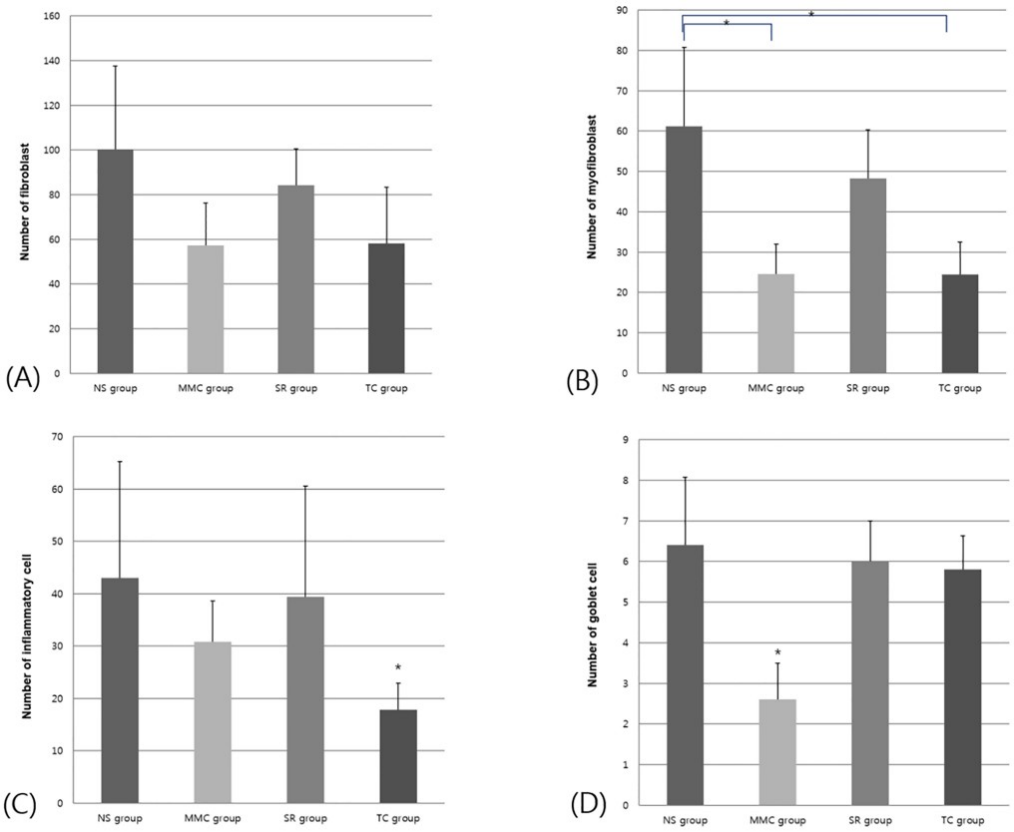
Histological outcomes of the conjunctiva and Tenon’s capsule. (A) The number of fibroblast showed no significant difference among the four groups. (B) The number of myofibroblasts significantly reduced in the MMC and TC groups. (C) The TC group had the lowest number of inflammatory cells among all groups (D) and the MMC group had the lowest number of goblet cells among all groups. NS = 0.9% normal saline, MMC = 0.5% mitomycin C, SR = sirolimus, TC = tacrolimus. * = significant difference in conjunctival congestion at 1 week among all groups (*p* < 0.05, Kruskal–Wallis test and Games–Howell adjustment).

**Fig 7:**
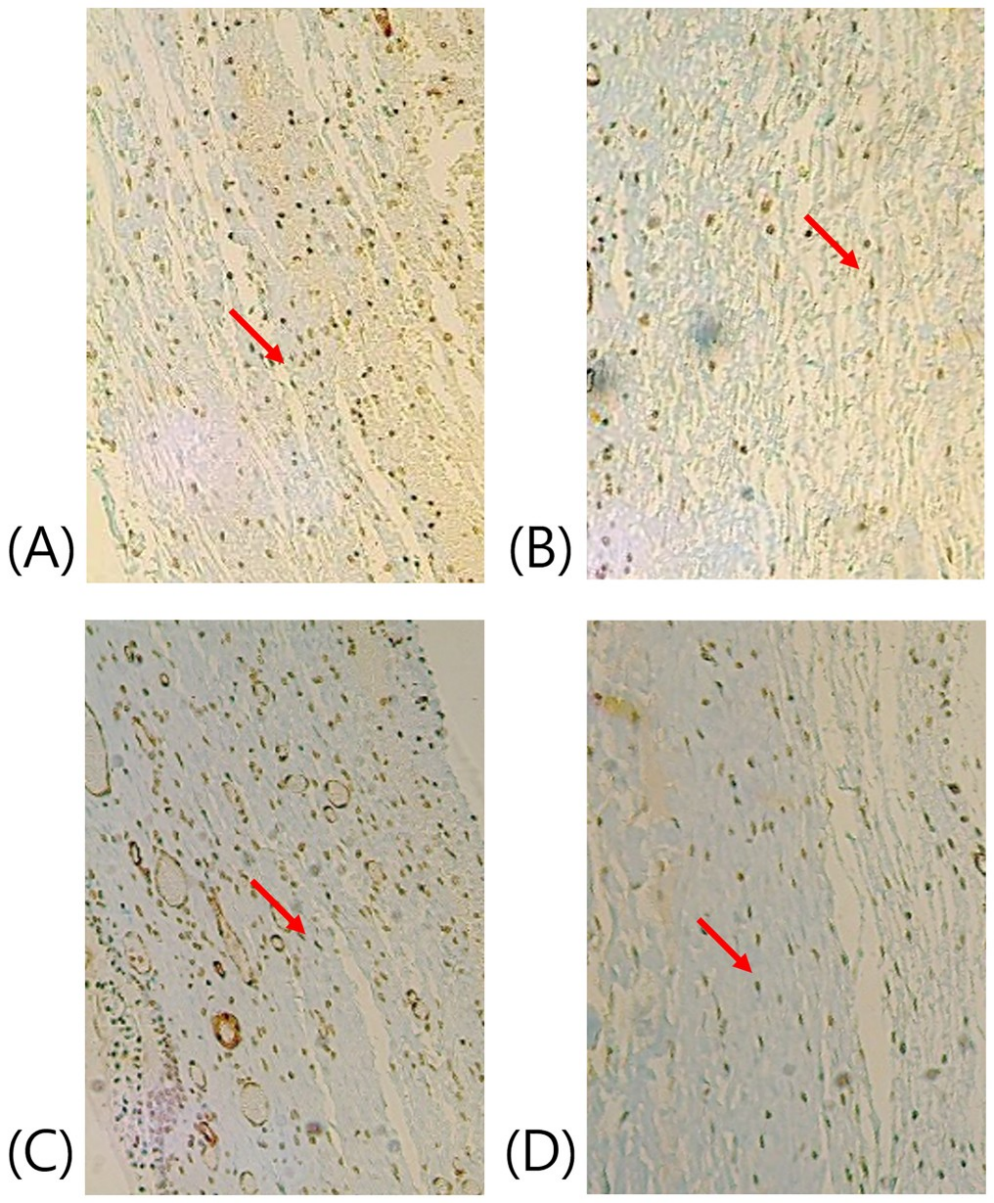
α- Smooth muscle actin staining results. (A) NS = 0.9% normal saline, (B) MMC = 0.5% mitomycin, (C) SR = sirolimus, and (D) TC = tacrolimus. (B and D) The number of myofibroblasts with high α-smooth muscle actin expression reduced in the MMC and TC groups (Light microscopy, 100×).

**Fig 8:**
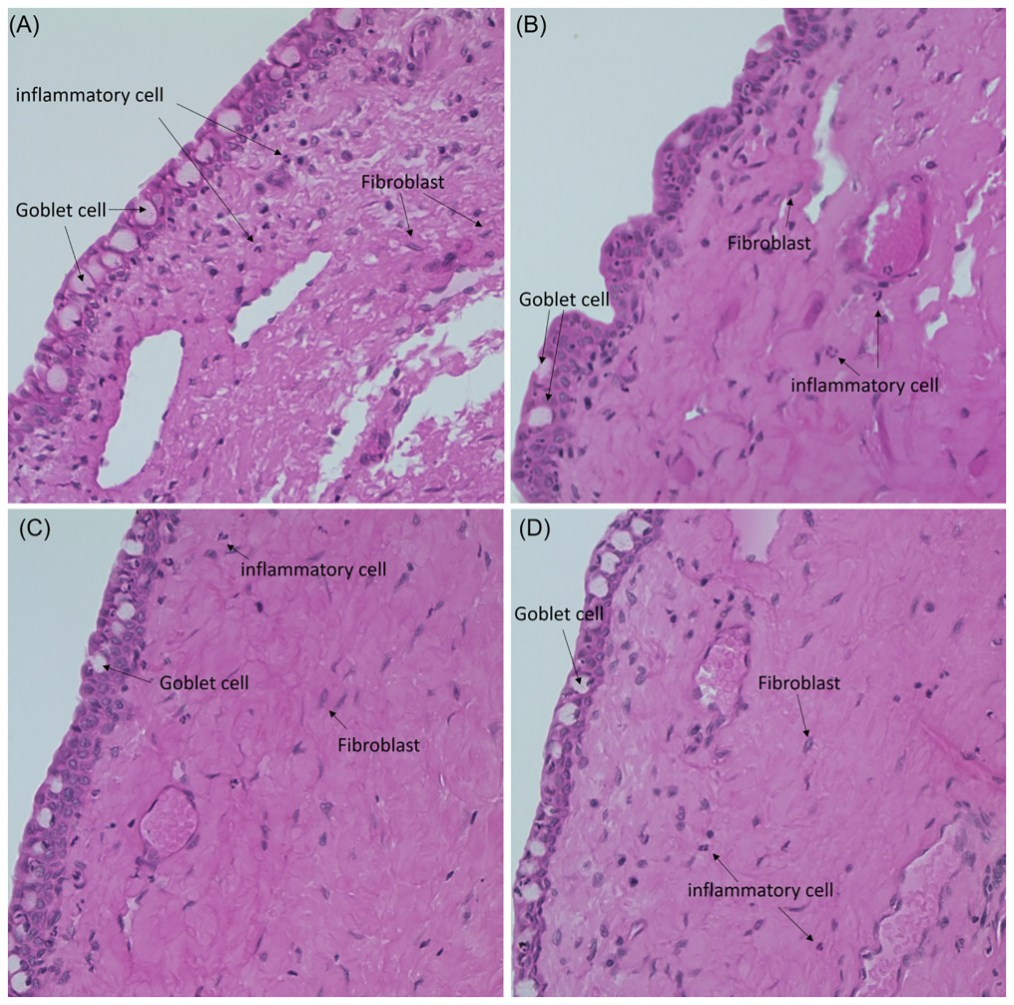
Representative images following H&E staining. (A) NS = 0.9% normal saline, (B) MMC = 0.5% mitomycin, (C) SR = sirolimus, and (D) TC = tacrolimus (Light microscopy, 400×).

The histological analysis showed that the number of fibroblasts was not significantly different between the four groups (Fig 6A). The TC group showed the lowest number of inflammatory cells among all groups (*p* < 0.05; Fig 6C). α-Smooth muscle actin (α-SMA) expression is a hallmark of mature myofibroblasts, which are brown stained cells (Fig 7, red arrow). The number of myofibroblasts with high α-SMA expression reduced in the MMC and TC groups (Figs 6B, 7B and 7D). Further, the MMC group had the lowest number of goblet cells among all groups (*p* < 0.05; Figs 6D and 8B).

The other histological findings did not differ significantly among the groups. In terms of postoperative complications, paracentral corneal opacity was observed at 1 week in one eye in the MMC group (S2 Fig).

## Discussion

In this study, we investigated the use of SR and TC as an alternative to MMC. These are macrolide antibiotics, which are frequently used as immunosuppressants. Since Salas-Prato et al. revealed that SR inhibits platelet-derived growth factor- and basic fibroblast growth factor-induced proliferation of human Tenon’s fibroblasts *in vitro*, the antifibrotic effect of SR was demonstrated in several studies [18–20]. Therefore, SR has also been frequently used as a coating drug in coronary stents to prevent restenosis of coronary arteries. However, only a few studies have investigated the effectiveness of SR against ocular disease [21, 22]. Ocular tolerability and efficacy of intravitreal and subconjunctival injections of SR have been reported in noninfectious uveitis patients (off-label use) [23]. Intravitreal SR injection—currently undergoing phase 3 trials in uveitis and other inflammatory pathways—has also been proposed as a suitable therapeutic option [24]. TC, which has shown a more potent immunosuppressive effect than cyclosporine A has often been used as an intravenous or oral agent for the treatment of myasthenia gravis [25, 26]. TC ointment is occasionally used for the treatment of refractory inflammatory ocular surface disease including atopic keratoconjunctivitis, vernal keratoconjunctivitis, and ocular graft-versus-host disease or for tapering steroids and preventing the recurrence of disease. However, its topical application is not widely used due to ocular discomfort (i.e., burning sensation and pruritus) [27–29].

TC exerts strong immunosuppressive effects. However, its use should be carefully monitored (i.e., concentration, dose, and treatment duration) due to severe drug toxicities including nephrotoxicity, hepatotoxicity, lung injury and carcinogenesis [30–33]. However, the drug-coated silicone plate used in the present study was isolated at the sub-Tenon’s space; thus, less discomfort than that reported with the use of topical TC ointment and minimal systemic effects were anticipated.

The early bare-metal stents prevented sudden arterial closure and reduced the possibility of restenosis compared with balloon angioplasty [34]. However, they were limited by the frequent occurrence of restenosis owing to smooth muscle proliferation and the resultant neo-intimal hyperplasia and target lesion revascularization [34]. Since the early 2000s, drug-eluting stents have been developed to prevent these effects. The implantation of SR-coated stent to narrowed coronary arteries presented short- and long-term success in drastically reducing the rates of coronary restenosis without the occurrence of systemic adverse effects in several large-scale clinical studies including the Randomized Study with the Sirolimus-Coated Bx Velocity Balloon-Expandable Stent (RAVEL study) [35–37]. The SR-coated stent prevented the formation of pseudomembrane by inhibiting the proliferation and migration of myofibroblasts. The SR was added in the sustained SR-releasing stent using two layers; one was a 5 μmol/L mixture layer of SR and synthetic polymer added to the inner surface of the stent (proximal layer), whereas the other was a layer in which a drug-free diffusion barrier was added over the layer containing SR (distal layer). The SR-coated stent was designed to adjust the rate of drug release and exert a long-term effect [38]. Other drugs including TC, paclitaxel, and zotarolimus were applied to coronary artery stents, achieving positive effects [39]. In the present study, the animal model of conjunctival fibrosis has been established as a pilot study by inserting a simple flattened silicone plate at the sub-Tenon’s space of the rabbits prior to the development of preliminary drug-eluting devices in ophthalmology. This slow-releasing coating technology can be used in glaucoma drainage implants to prevent stenosis due to fibrosis. Drug-eluting implants, subconjunctivally placed adjacent to the surgical site immediately prior to conjunctival closure, exert long-acting local beneficial effect and are linked to a low incidence of systemic adverse effects compared with topical or subconjunctival administration [40].

In this study, the degree of conjunctival congestion in the MMC group was more prominent, and the number of goblet cells was the lowest among all groups (S1 Fig and Fig 6D). Furthermore, paracentral corneal opacity was observed in one eye in the MMC group; however, severe adverse effects (e.g., tissue necrosis) did not occur (S2 Fig). This finding indicates that the toxicity of MMC was higher than those observed for the other drugs. Considering that drug-eluting stents continuously release drugs for a long period, and MMC-related complications occur in a dose- and time-dependent manner, MMC is more suitable for short-term use rather than a good candidate for drug-eluting stents.

In the TC group, conjunctival hyperemia was significantly reduced from 1 week postoperatively. The SR and TC groups showed a significant reduction in conjunctival hyperemia at 4 weeks compared with 1 week (S1 Fig). At 4 weeks, similar conjunctival hyperemia was observed between the two groups (S1 Fig). Although histological findings were not confirmed at 1 week, these results suggest that the TC-coated silicone plate exerted a relatively faster effect.

The histological analysis showed that the number of goblet cells in the SR and TC groups was similar to that observed in the NS group (Fig 6D). This result indicates that SR and TC were less damaging to healthy conjunctival tissue. Collectively, the TC-coated silicone plate administered in the sub-Tenon’s space effectively inhibited inflammation and fibrosis without the occurrence of significant adverse effects at 4 weeks.

In general, the concentration of MMC, SR, and TC in topical or subconjunctival administration is approximately 0.2–0.5 mg/mL [41–43]. In another study, the concentration of MMC applied in the production of poly(lactic-co-glycolic acid) implants, as well as on their coating, was 1.25 mg/mL [40]. However, we had to use a higher concentration of drug than that used in previous studies to coat silicon rubber. It was necessary to increase the concentration of the coating drug because the amount of drug released at the drug-coated plate was much lower than that of either drug instillation or injection. The coating concentration of each drug was finally determined *in vitro* using liquid chromatographic-tandem mass spectrometry (Figs 3 and 4).

Recently, the TiO_2_ nano-coating drug delivery system was successfully used as a sustained growth factor-releasing system for dental implants [44]. However, to the best of our knowledge, this is the first study to investigate the application of the TiO_2_ nano-coating drug delivery system on a silicon plate for intraocular use.

This study had several limitations. Firstly, the study included a low number of subjects and relatively short follow-up period (1 month). To better determine the expression of myofibroblasts, it is necessary to perform a study with a 2–3 month follow-up period [45]. Secondly, the pharmacokinetics and pharmacodynamics of the drug-coated silicon plate were not determined *in vivo*. Further studies are warranted to determine the optimal concentration of the drug in the coating agent by measuring the concentration of aqueous humor throughout a prolonged period.

In conclusion, among the drugs used in this study, TC was the most effective in inhibiting inflammation and myofibroblasts and was associated with the lowest incidence of adverse effects in the rabbit model. The TC-coated silicone plate can be used as an ophthalmic drug-coated implant for the inhibition of fibrosis. Tacrolimus may prove to be an effective alternative to mitomycin C.

## Supporting information

S1 Fig The photographs of conjunctiva around the inserted drug-coated silicon plate. (A) NS = 0.9% normal saline (B) MMC = 0.5% mitomycin (C) SR = sirolimus (D) TC = tacrolimus; 1w = 1 week; 4w = 4 weeks.
(TIF)

S2 Fig Paracentral corneal opacity of the mitomycin C group. (TIF)

S1 Table Histologic data of four groups. (DOCX)

S1 Dataset Dataset containing the scores of conjunctival congestion and histologic data of four groups. (XLSX)
