# Correction: Safety and efficacy of tacrolimus-coated silicone plates as an alternative to mitomycin C in a rabbit model of conjunctival fibrosis

**DOI:** 10.1371/journal.pone.0226662

**Published:** 2019-12-12

**Authors:** Sam Young Yoon, Eun-Soon Kim, Gill Sang Han, Leejee H. Suh, Hyun Suk Jung, Hungwon Tchah, Jae Yong Kim

In the Funding statement, the grant number from the funder Ministry of Education, Science and Technology (MEST) is listed incorrectly. The correct grant number is: NRF-2018R1D1A1B07043010.
