# Supplementary figures and images for: Safety and efficacy of tacrolimus-coated silicone plates as an alternative to mitomycin C in a rabbit model of conjunctival fibrosis
Source: PLoS One. 2019 Jul 5;14(7):e0219194. doi: 10.1371/journal.pone.0219194 (PMC6611608; doi:10.1371/journal.pone.0219194)

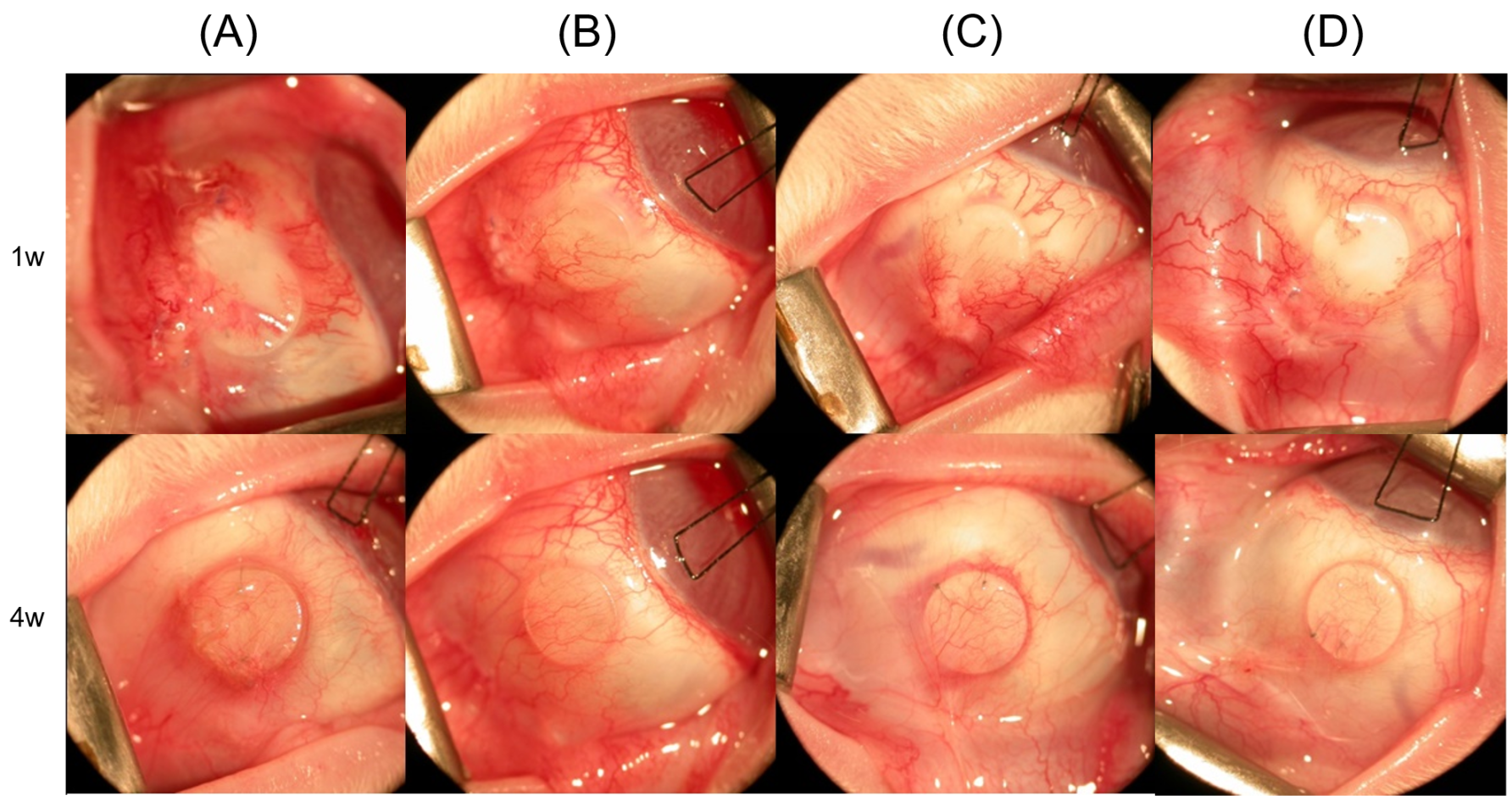

Supplement: S1 Fig — (A) NS = 0.9% normal saline (B) MMC = 0.5% mitomycin (C) SR = sirolimus (D) TC = tacrolimus; 1w = 1 week; 4w = 4 weeks. (TIF) [file pone.0219194.s001.tif]

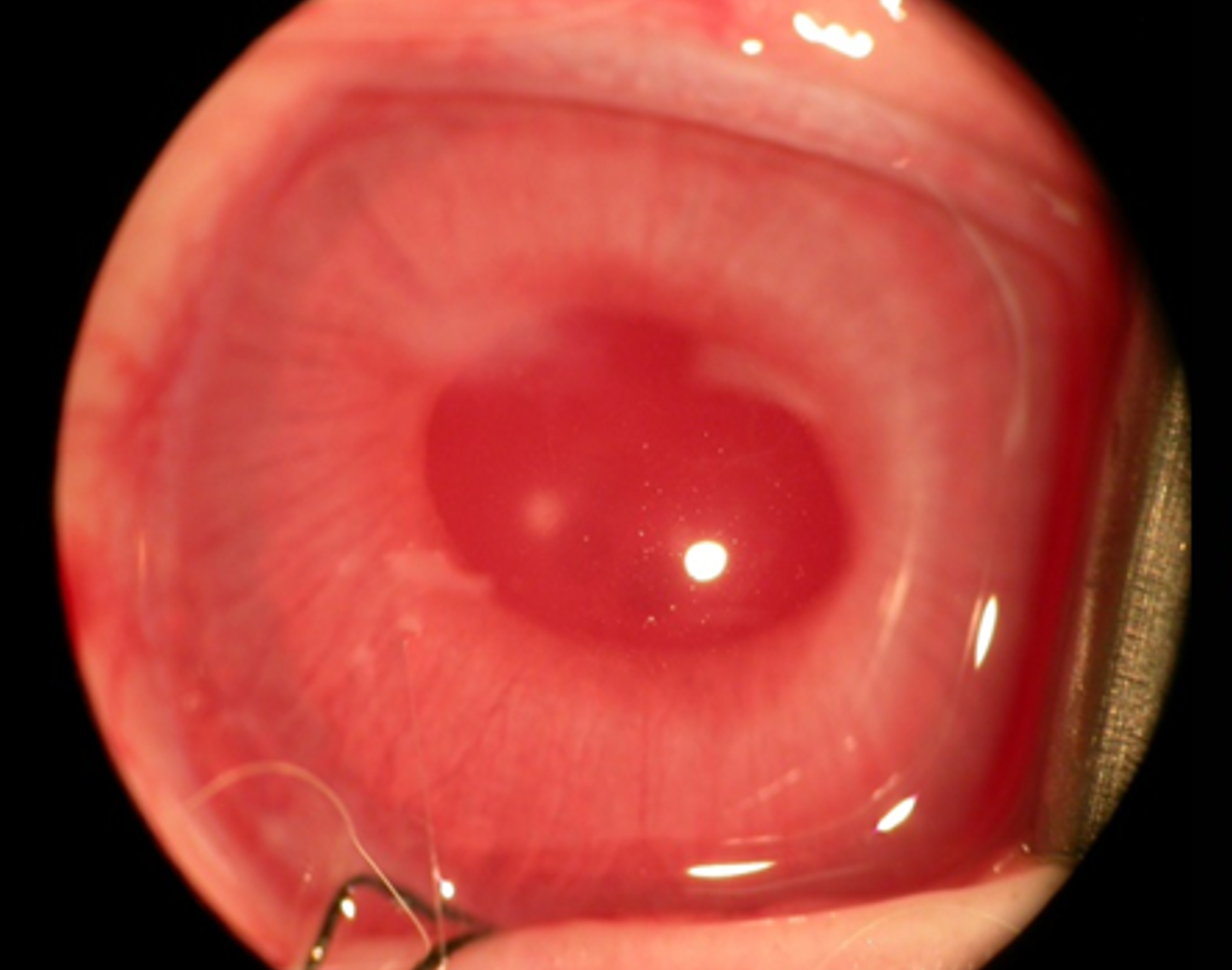

Supplement: S2 Fig — (TIF) [file pone.0219194.s002.tif]
