# Supplementary material for: Safety and efficacy of tacrolimus-coated silicone plates as an alternative to mitomycin C in a rabbit model of conjunctival fibrosis
Source: PLoS One. 2019 Jul 5;14(7):e0219194. doi: 10.1371/journal.pone.0219194 (PMC6611608; doi:10.1371/journal.pone.0219194)
Supplement: S1 Table — (DOCX) [file pone.0219194.s003.docx]

**S1 Table. Histologic data of 4 groups**

|  | fibroblast | myofibroblast | inflammatory cell | goblet cell |
| --- | --- | --- | --- | --- |
| NS | 100.2 ± 33.5 | 61.2 ± 17.5 | 43.0 ± 19.9 | 6.3 ± 1.2 |
| MMC | 57.2 ± 17.0 | 24.7 ± 6.6 | 30.8 ± 7.0 | 2.5 ± 0.5 |
| SR | 84.2 ± 14.7 | 48.2 ± 10.9 | 39.5 ± 18.8 | 6.0 ± 0.9 |
| TC | 58.2 ± 22.4 | 24.3 ± 7.2 | 17.8 ± 4.5 | 5.8 ± 0.8 |
| Mean ± SD, cell count  NS = 0.9% normal saline; MMC = 0.5% mitomycin; SR = sirolimus; TC = tacrolimus. | | | | |
